# Use of Argon Plasma Coagulation and Endoscopic Hemoclips for Postsurgical Gastrointestinal Fistula

**DOI:** 10.1155/2020/8835437

**Published:** 2020-11-11

**Authors:** Abdullah Sohail, Ahmad Khan, Hamza Shah, Ragheb Rezko

**Affiliations:** ^1^West Virginia University Charleston Division, Charleston, West Virginia, USA; ^2^Charleston Area Medical Center, Charleston, West Virginia, USA; ^3^West Virginia University, Morgantown, West Virginia, USA

## Abstract

A 72-year-old male was diagnosed with a duodenal mass and underwent extensive surgical resection. The patient's post-op course was complicated by an anastomotic leak that was first treated conservatively; however, his condition continued to deteriorate. An upper endoscopy was performed, which showed misplacement of drain forming a fistulous track through the lumen of the bowel. We removed the drain and used argon plasma coagulation to de-epithelize the lumen and closed the fistula with hemostasis clips. The patient's clinical status improved significantly. Our case emphasizes the success of endoscopic techniques as an alternative option in the management of postsurgical anastomotic leaks and fistulas in the right clinical setting and patient population.

## 1. Introduction

Postsurgical anastomotic leaks and fistulas are a serious complication after major abdominal surgery resulting in significant morbidity and mortality [1]. Its occurrences depend on surgical procedure type, technique, expertise, and anatomical location [2]. The estimated incidence is reported between 4% to 17% in surgical procedures involving the upper gastrointestinal (GI) tract and 2% to 7% in colorectal surgeries. Similarly, associated mortality is described 10% to 65% in the upper GI tract and 6% to 22% in lower GI tract surgeries [3, 4].

Historically, the mainstay of treatment has been either conservative management such as prolonged bowel rest with total parenteral nutrition (TPN) or surgical interventions. Lately, endoscopic treatments have emerged as an alternative option for the management of anastomotic leaks/fistulas with better clinical outcomes [5].

We present a case of a postsurgical anastomotic leak and enterocutaneous fistula formation after an extensive small bowel resection who deteriorated with conservative/surgical interventions and had successful outcomes with endoscopic treatment.

## 2. Case Report

**Figure 1:**
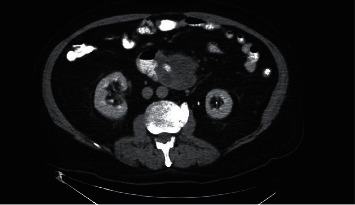
Initial CT scan showing mass in the duodenum.

**Figure 2:**
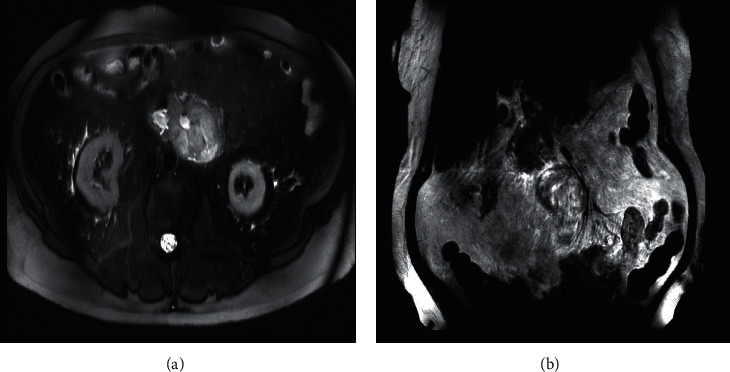
MRI abdomen confirming duodenal mass in both (a) sagittal and (b) coronal sections.

**Figure 3:**
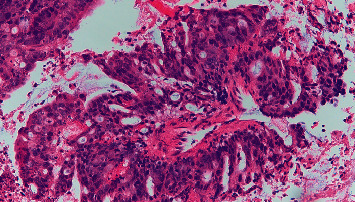
Invasive moderately differentiated adenocarcinoma with extracellular mucin production.

**Figure 4:**
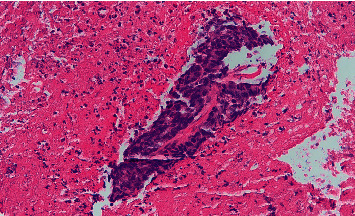
A group of malignant columnar shaped epithelioid cells in a background of abundant necroinflammatory debris.

**Figure 5:**
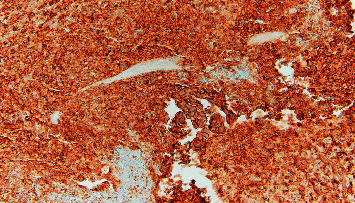
Malignant cells are positive for MOC-31 stain.

**Figure 6:**
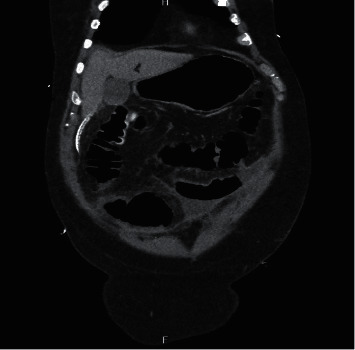
CT abdomen showing extravasation of contrast material.

**Figure 7:**
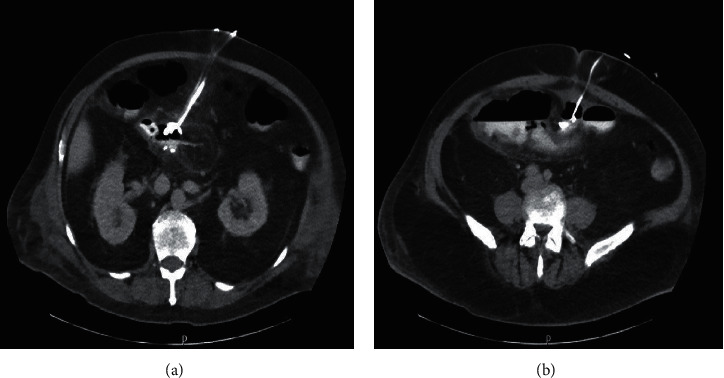
CT showing drainage catheters at the anastomotic site.

**Figure 8:**
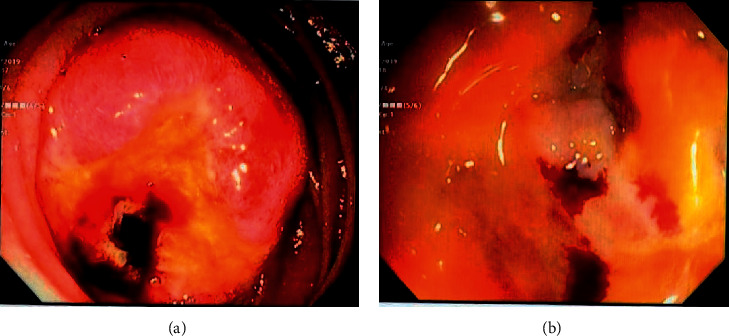
Endoscopic images of misplaced drainage catheter in the lumen of the duodenum.

**Figure 9:**
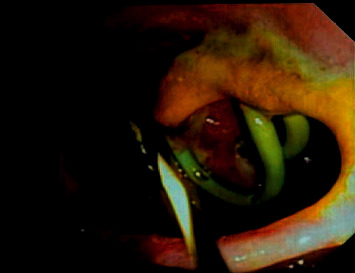
Drainage catheter in the lumen of the duodenum.

**Figure 10:**
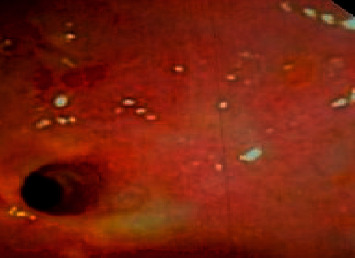
Fistulous tract opening in the lumen.

**Figure 11:**
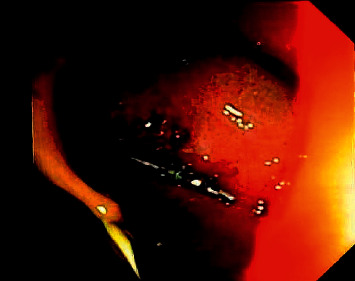
Closure of the tract by placing hemoclips.

**Figure 12:**
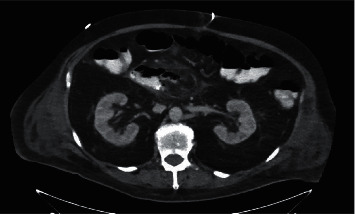
Follow-up CT scan showing resolution of anastomotic leakage.

A 72-year-old male presented to the emergency department with complaints of four-day history of epigastric abdominal pain, nausea, vomiting, constipation, and inability to tolerate oral diet. He reported early satiety and 24 pounds of unintentional weight loss in the last four months. He was hemodynamical stable on presentation. Abdominal examination revealed mild epigastric tenderness with moderate abdominal distention. The basic laboratory workup was within normal limits. However, computed tomography (CT) abdomen and pelvis with intravenous and oral contrast showed eccentric thickening of the third and fourth part of the duodenum (Figure 1). A magnetic resonance imaging showed a heterogeneous mass in the duodenum measuring 13.1 × 5.9 × 7.5 cm (Figure 2). An esophagogastroduodenoscopy (EGD) was performed to obtain the biopsy of the mass which turned out to be adenocarcinoma of the small bowel (Figures 3–5). He underwent open distal duodenectomy with extensive small bowel resection. His postoperative course was complicated by fever, hypotension, tachycardia, and persistent abdominal pain. A follow-up CT scan of the abdomen revealed an anastomotic leak at the surgical site (Figure 6). Although the patient was started on complete bowel rest, TPN, and broad-spectrum antibiotics, his clinical condition continued to deteriorate. Two 8 French pigtail drainage catheters were placed around the anastomosis leak which continued to have high output over the next few days (Figure 7). A follow-up CT revealed a persistent anastomotic leak with the formation of an enterocutaneous fistula involving the duodenum. A repeat EGD was performed, and the tip of one of the catheter was found in the lumen of the duodenum forming a fistulous tract (Figures 8–10). The catheter was removed, and we applied argon plasma coagulation (APC) through the fistulous tract and around the edges to allow de-epithelialization and granulation tissue formation. The closure of the tract was achieved by placing over the scope hemoclips (Figure 11). A follow-up CT scan of the abdomen and pelvis showed resolution of the anastomotic leak (Figure 12). The patient's clinical condition markedly improved and was transferred out of the ICU and later discharged home.

## 3. Discussion

The endoscopic management of postsurgical intestinal anastomotic leaks and fistulas serves as an alternative to conservative and surgical treatment options. These techniques have the advantage of being minimally invasive that results in a shorter length of stay and antibiotic therapy. Different endoscopic modalities used have unique mechanisms and are used in combination or alone [6].

The use of self-expandable metal stents (SEMS) allows the diversion of the gastrointestinal contents away from the site of leakage to provide a stable environment for healing [7, 8]. Extraction of fully covered SEMS is easier than partially or uncovered SEMS which makes these preferred over the others. Also, the latter become rapidly embedded in the mucosa due to tissue hyperplasia that makes them less favorable.

Hemostatic clips including through the scope (TTS) clips and over the scope (OTS) clips are used in closing the GI defects [9]. TTS clips have a disadvantage of a weak and superficial grasp and hence are less preferred and also OTS provides better pressure for tissue opposition. Haito-Chavez et al. reported a series of 188 patients who underwent management with OTS with an overall success rate of 90% and 73.3% for intestinal perforations and leaks, respectively [10].

Endoscopic suturing devices can also be used in the management of GI defects depending upon the location and type of the lesions. The Overstitch device (Apollo Endosurgery) is the most commonly used device that helps to apply sutures in a continuous or interrupted fashion. Sharaiha et al. reported the results from a large multicenter study for comprising of 122 patients who underwent endoscopic suturing to anchor stents (*n* = 47; 38.5%), fistulas (*n* = 40; 32.7%), leaks (*n* = 15; 12.3%), and perforations (*n* = 20; 16.4%). The immediate clinical success was ascertained in 79.5%, and long-term clinical success was noted in 78.8% after a mean follow-up of 68 days [11].

The vacuum-assisted closure (VAC) device is another promising option to be considered. In this technique, a vacuum-sealed sponge connected to a constant suction is inserted into the wound cavity. This helps in the removal of fluid and debris, promoting granulation tissue. Wedemeyer et al. successfully treated 8 patients with postesophagectomy leaks with VAC. The leak got healed in 7 out of 8 patients (88%) with a mean duration of 23 days [12].

One of the major issues in the postsurgical leak healing process is the epithelialization of the defect and subsequent tract formation which results in the formation of chronic draining fistulas. This usually slows down the healing process and limits the use of other endoscopic therapies. Therefore, de-epithelialization of the leak edges is vital in these cases and can be achieved by Argon Plasma coagulation (APC) as one of the preferred methods [13, 14]. The possible adverse effects of APC include perforation, pneumoperitoneum, and subcutaneous bubbling of gas [15, 16].

Postsurgical anastomotic leaks have serious complications with significant morbidity and mortality. As mentioned, there are several endoscopic techniques which have shown efficacy and promising outcomes with no single standard technique to manage individual defects, rather a combination of techniques, complementary to each other. The choice of the endoscopic technique depends on several factors including the site, size, duration of the leak, and operator experience with these techniques. In our patient, we used APC to de-epithelize or denude the luminal surface of the involved intestinal tract and later used hemostatic clips. The hemostatic clip was used since the size of the defect was small. Our case emphasizes the use of endoscopic techniques to treat postsurgical anastomotic leaks and fistulas early on in suitable patients as a successful alternative option to conventional therapies; however, future large case series or prospective studies are required to validate the efficacy and safety of endoscopic therapies.
